# Production of a Recombinant Single-Domain Antibody for Gluten Detection in Foods Using the *Pichia pastoris* Expression System

**DOI:** 10.3390/foods9121838

**Published:** 2020-12-10

**Authors:** Aina García-García, Raquel Madrid, Eduardo Garcia-Calvo, Belén Mendoza-Chamizo, Teresa García, Rosario Martin

**Affiliations:** Departamento de Nutrición y Ciencia de los Alimentos, Facultad de Veterinaria, Universidad Complutense de Madrid, 28040 Madrid, Spain; raqmad01@ucm.es (R.M.); edugar01@ucm.es (E.G.-C.); bemendoz@ucm.es (B.M.-C.); tgarcia@ucm.es (T.G.); rmartins@ucm.es (R.M.)

**Keywords:** domain antibody, gluten, enzyme-linked immunosorbent assay (ELISA), recombinant antibody, *Pichia pastoris*, food allergen detection, food analysis

## Abstract

The detection of gluten in foodstuffs has become a growing concern in food allergen management as a result of the high ratio of population sensitive to the main gluten-containing cereals. In this study, a promising single-domain antibody previously isolated by phage display (dAb8E) was produced in *Pichia pastoris* resulting in high levels of the antibody fragment expression (330 mg/L). The purified dAb8E was proved to specifically bind to gluten proteins from wheat, barley and rye, exhibiting no cross reaction to other heterologous species. The dynamic range of the sandwich enzyme-linked immunosorbent assay (ELISA) covered 0.1 to 10 µg/mL of gliadin, reaching a limit of detection of 0.12 µg/mL. When experimental binary mixtures of the target cereals were analyzed, the limit of detection was 0.13 mg/g, which would theoretically correspond to gluten concentrations of approximately 13 mg/kg. Finally, thirty commercially available food products were analyzed by means of the developed assay to further confirm the applicability of the dAb8E for gluten determination. The proposed methodology enabled the generation of a new gluten-specific nanobody which could be used to guarantee the appropriate labelling of gluten-free foods.

## 1. Introduction

Adverse reactions to food, which affect a significant percentage of the world population, have become a major economic and health concern because they are responsible for a significant number of hospitalizations and food recalls [1]. Specifically, the ingestion of gluten-containing cereals can trigger an array of different conditions, collectively designated as “gluten-related disorders”, that include different autoimmune disorders (i.e., coeliac disease, dermatitis herpetiformis and gluten ataxia), wheat allergy and the controversial non-coeliac gluten sensitivity [2,3]. In all cases, the only effective treatment for patients suffering from these pathologies is the complete withdrawal of the harmful ingredient from the diet [4]. Therefore, the development of methods that allow detection and quantitative determination of the gluten proteins have gained importance in the last decades [5,6].

Different approaches, including immunodetection, liquid chromatography coupled to mass spectrometry and polymerase chain reaction (PCR)-based methodologies, have been extensively used for the detection of food allergens [7]. Regarding the detection of gluten, enzyme-linked immunosorbent assay (ELISA) and lateral flow devices have been adopted as official methods to ensure the enforcement of food labelling regulations [6,8]. In this context, the European Union Regulation (EU) No. 828/2014 defines that a food product labelled as “gluten-free” may not exceed the maximum safety threshold established at 20 mg/kg of gluten [9]. Nevertheless, large discrepancies in the determination of gluten depending on to the selected immunoassay and the gluten extraction protocol have been widely reported [10,11].

Since the discovery of fully functional naturally occurring antibodies which lack the light chains, the single-domain antibody fragments (sdAbs) have been shown to possess privileged access to distinct antigenic regions on proteins due to its small dimension in comparison to a conventional antibodies [12,13]. These antibody fragments composed solely of the heavy chain variable domain, also referred to as nanobodies, has demonstrated its remarkable diagnostic potential in a wide range of applications [13,14]. In this regard, different researches have demonstrated the benefits of sdAbs in food allergens detection [15,16,17,18].

Different hosts have been used for the production of recombinant sdAbs, such as yeast, mammalian and bacterial cells [19]. The methylotrophic yeast *Pichia pastoris* (*Komagataella phaffi*) has emerged as one of the most effective production systems of both secreted and intracellular recombinant proteins. Initially chosen for their inexpensive and efficient growth in methanol-containing media, this eukaryotic single-cell factory has currently demonstrated important benefits [20,21]. *P. pastoris* is capable to perform post-translational modifications such as glycosylation, disulfide bridge formation or proteolytic processing, which result in correctly folded proteins secreted into culture media, thus simplifying the purification stage due to the small presence of other native proteins. Regarding the production of recombinant antibodies, this yeast is one of the most established organisms for the expression of single-chain variable fragment (scFv) and scFv-fusion proteins [22,23], whereas the production of nanobodies in *P. pastoris* is a less established approach that still needs to be explored [24,25,26].

In previous studies, we demonstrated the feasibility of isolating a gluten-specific single-domain antibody from a semisynthetic library by phage display, thus avoiding animal immunization [18]. The present work aims to produce in a soluble form the antibody selected as the best candidate in order to be used as a novel bio-recognition molecule for the analysis of gluten in foods. For this purpose, the yeast *P. pastoris* was proposed as the expression system and the applicability of the purified antibody fragment (dAb8E) in ELISA was assessed.

## 2. Materials and Methods

### 2.1. Materials and Chemicals

*Escherichia coli* DH5α strain [*fhuA2 lac(del)U169 phoA glnV44 Φ80′ lacZ(del)M15 gyrA96 recA1 relA1 endA1 thi-1 hsdR17*] (Invitrogen, Carlsbad, CA, USA) was used for plasmid construction and amplification and *Pichia pastoris* X-33 strain (Life Technologies, Carlsbad, CA, USA) was used for recombinant antibody expression. Restriction enzymes *Pst*I, *Not*I and *Pme*I were purchased from New England Biolabs (Hitchin, UK). Calf intestinal alkaline phosphatase, T4 DNA Ligase and GoTaq^®^ Green Master Mix were purchased from Promega (Madison, WI, USA). Purification kits (QIAGEN Plasmid Midi Kit and QIAquick Gel Extraction Kit) were purchased from Qiagen (Hilden, Germany). Unless otherwise stated, remaining chemicals and synthetic oligonucleotides were purchased from Sigma-Aldrich (St. Louis, MO, USA).

Bacteria were grown in low-salt Luria Bernati broth (LB: 5 g/L NaCl, 5 g/L yeast extract and 10 g/L tryptone, pH 7.5. Additionally, 15 g/L agar was added for making LB plates). Super optimal broth with catabolite repression (SOC) medium, used to aid recovery of bacterial competent cells after transformation, was 5 g/L yeast extract, 20 g/L tryptone, 10 mM NaCl, 2.5 mM KCl, 10 mM MgCl_2_, 10 mM MgSO_4_ and 20 mM glucose. *P. pastoris* was cultured in yeast extract peptone dextrose (YPD) medium (10 g/L yeast extract, 20 g/L peptone, 20 g/L dextrose. Additionally, 20 g/L agar was added to YPD plates and 1 M sorbitol to YPDS). The Buffered Minimal Glycerol-complex Medium (BMGY) was prepared with 10 g/L yeast extract, 20 g/L peptone, 1% glycerol, 1.34% yeast nitrogen base (YNB) with ammonium sulphate and without amino acids, and 4 × 10^−5^% biotin in 100 mM potassium phosphate, pH 6.0. Methanol (Fisher Scientific, Loughborough, UK) was used for *P. pastoris* induction in Buffered Minimal Methanol-complex Medium (BMMY), prepared as BMGY except 1% methanol replaced 1% glycerol as sole carbon source. Selection antibiotic Zeocin^TM^ was purchased from Life Technologies.

The Spanish National Center for Plant Genetic Resources (CRF-INIA, Madrid, Spain) kindly supplied different kernels of common, spelt, rivet and durum wheats, barley and rye (10 cultivars each). More information about these reference samples can be obtained from García-García et al. 2019 [27]. Non-target plant species included in the specificity assays and 30 commercially available foodstuffs from several brands were acquired from local retailers and food stores in Madrid (Spain) including products with different declarations with regard to the gluten-containing cereals.

### 2.2. Vector Construction

Vector pMJA302 which contains the nucleotide sequence encoding the gluten-specific antibody fragment dAb8E, was constructed as follows: *Escherichia coli* TG1 clones infected with the selected phage-dAbs were grown overnight into 20 mL of 2xTY (supplemented with 100 μg/mL ampicillin and 40 g/L glucose) to subsequently purify the phagemid pR2 vector of interest. Primers MJA448 (TAT TAT AT*C TGC AG*A GGT GCA GCT GTT GGA GTC TGG G) and MJA449 (TAT TAT ATG *GCG GCC GCG* CTC GAG ACG GTG ACC AGG GTT) were used to amplify the antibody fragment coding sequence incorporating respectively the restriction site for *Pst*I and *Not*I (in italics). Gel purified PCR product and acceptor plasmid pMJA282 (modified from the commercially available pPICzαB to enlarge the multiple cloning site) were digested with *Pst*I and *Not*I for 3 h at 37 °C and incubated for 20 min at 65 °C for inactivation of the enzymes. After gel purification of the digestions, insert and vector were ligated in a 3:1 ratio using the T4 DNA ligase for 2 h at 25 °C and transformed into *E. coli* competent cells.

### 2.3. Transformation of Escherichia coli

The ligation reaction was mixed with 40 µL of freshly made calcium competent cells (see Appendix A.1 for complete procedure) and the following temperature program was run: 2 min at 4 °C, 30 s at 42 °C and 10 min at 4 °C. Subsequently, 100 µL of SOC medium was added and incubated at 37 °C for 60 min with shaking. The transformation was spread in pre-warmed LB agar plates supplemented with 25 µg/mL of Zeocin^TM^ and incubated at 37 °C overnight.

The positively transformed clones were selected by colony PCR and confirmed by the digestion pattern of the purified plasmids. Finally, the correct sequence identity and orientation was confirmed by DNA sequencing with primer MJA366 (TGC ATC TCT CAG GCA AAT GGC A).

### 2.4. Transformation and Induction of Pichia pastoris

The expression vector pMJA302 was purified from an overnight culture of a positively transformed *E. coli* clone and quantified with a NanoDrop ND-1000 spectrophotometer (NanoDrop Technologies Inc., Montchanin, DE, USA). A total of 20 µg of vector was linearized by *Pme*I digestion for 3 h at 37 °C, ethanol precipitated and transformed into electrocompetent *P. pastoris* X-33 cells following the method described by Cregg and Russell (1998) [28] (see Appendix A.2 for complete procedure). In a sterile microfuge tube, 10 µg of the linearized plasmid and 50 µL of the electrocompetent cells were mixed and incubated on ice for 1 min. The sample was transferred to pre-chilled 0.2 cm electroporation cuvettes (BioRad, Hercules, CA, USA) and transformed with a BioRad MicroPulser electroporation apparatus, according to the parameters for *P. pastoris* as suggested by the manufacturer (2000 V, 25 μF, 200 Ohm). After pulsing, the cells were immediately recovered in 1 mL ice-cold yeast extract peptone dextrose medium with sorbitol (YPDS) and incubated at 30 °C for 2 h without shaking. Finally, the transformed cells were spread in YPDS agar plates containing 300 μg/mL Zeocin^TM^ and incubated at 30 °C for 72 h for the selection of positive transformants.

Five isolated colonies were screened for recombinant protein production. Firstly, PCR analysis of the clones was performed to verify that the recombinant plasmids were correctly transformed and integrated into the genome of *P. pastoris* employing the set of primers MJA366/MJA448. Subsequently, the selected clones were grown into 150 µL of YPDS with 300 μg/mL Zeocin^TM^, and incubated overnight at 30 °C with shaking (200 rpm) in a 96-well round-bottom microwell plate (Costar, Corning Life Sciences, New York, NY, USA). Next day, 1.5 mL of BMGY with 300 μg/mL Zeocin^TM^ was inoculated with 30 µL of each isolated clone in 24 well plates. Plates were incubated at 30 °C with shaking until next day, centrifuged at 1800× *g* for 15 min at 4 °C and the supernatants removed by aspiration with a vacuum pump. To induce protein expression, cells were resuspended in 1.5 mL BMMY and incubated at 26 °C for 72 h with addition of 1% (*v*/*v*) methanol every 24 h to maintain induction. Finally, the plates were centrifuged and the supernatants were analyzed to evaluate the expression levels of the heterologous protein.

### 2.5. Characterization of the Antibody Fragment dAb8E

#### 2.5.1. Analysis of the Expression in the Supernatant of *Pichia pastoris*

A PVDF membrane (Immun-Blot, BioRad) was coated through a dot blot microfiltration unit (Life Technologies) with one hundred microliters of the induced and non-induced supernatants of the five selected clones transformed with pMJA302 plasmid. PBS and yeast broth were used as negative controls and 10 µL of an antibody fragment previously purified in our group at 0.1 µg/µL was used as positive control (of almond-specific single chain antibody containing a c-myc epitope and hexa-histidine tag) [29]. The membrane was blocked overnight at 4 °C with 3% BSA (*w*/*v*) in TBS (50 mM Tris HCl, 150 mM NaCl, pH 7.5). After three washing steps with TBST (TBS with 0.05% (*v*/*v*) Tween‑20), the membrane was incubated for 2 h at 37 °C in a rocking platform with a 1:5000 (*v*/*v*) mouse monoclonal anti-c-myc antibody (Sigma Aldrich) solution in 1% BSA (*w*/*v*) TBST. After washing three times with TBST, the membrane was incubated in the same conditions with a 1:20,000 (*v*/*v*) goat-anti-mouse IgG-ALP antibody (Sigma Aldrich) solution in 1% BSA (*w*/*v*) TBST. Finally, the membrane was washed three times with TBST and rinsed with water before adding a ready to use solution of 5-bromo-4-chloro-3-indolyl-1-phosphate and nitroblue tetrazolium (Novex AP Chromogenic Substrate, Life Technologies). When the alkaline phosphatase enzyme activity was revealed, the reaction was stopped by rinsing the membrane with water.

The supernatants were complementarily analyzed by sodium dodecyl sulfate-polyacrylamide gel electrophoresis (SDS-PAGE) in a 12% resolving gel at 100 V using Mini-Protean Tetra Cell (Bio-Rad Laboratories, Hercules, CA, USA). Ten microliters of the induced yeast culture were diluted in Laemmli sample buffer (2×) containing 5% (*v*/*v*) 2-mercaptoethanol. Afterwards samples were heated for 10 min at 95 °C and centrifuged for 1 min at 8000× *g* before being loaded on the electrophoresis gel. Colorburst (8–220 kDa) was used as the protein molecular weight marker (Sigma) and Blue Safe (Nzytech, Lisbon, Portugal) as the staining solution.

Production of two selected clones (3 and 4) was scaled up to 150 mL. Growth and methanol induction conditions, as well as harvesting of the medium were similar as mentioned above but using a two-liter flask instead of the plate cultures. To assess recombinant protein production over time, supernatant samples were taken every 24 h, prior to the addition of methanol, and analyzed by dot-blotting. Briefly, a 10 µL drop of the 10-fold-diluted supernatants was allowed to dry on a PVDF membrane, washed three times with PBS and blocked with 2% skimmed milk powder in PBS (M‑PBS) for 2 h at room temperature. Subsequently, the membrane was incubated for 2 h at 37 °C in a rocking platform with a 1:5000 (*v*/*v*) anti-HisTag-HRP antibody (Abcam, Cambridge, UK) in blocking buffer. Finally, after washing three times with PBS, the membrane was revealed with the chemiluminescent substrate Clarity Western ECL (Bio-Rad). Prior to purification, the supernatants were additionally analyzed by SDS-PAGE, as mentioned above, and Western blot analysis by transferring an equivalent gel onto a PVDF membrane at 110 V for 1 h in an ice-water bath using the Mini Trans-Blot System (Bio-Rad Laboratories). After complete transfer, the membrane was revealed as previously described using the anti-HisTag-HRP antibody.

#### 2.5.2. Purification of the Recombinant Protein dAb8E

For further characterization of the recombinant dAb8E fragment, the resulting supernatant obtained after 72 h of methanol induction in the large-scale production was collected by centrifugation (3000× *g*, 5 min, 4 °C) and dialyzed against 20 mM potassium phosphate buffer (PBS, pH 7.5) for 18 h at 4 °C using a SnakeSkin dialysis tubing (10 kDa MWCO, 35 mm internal diameter) (ThermoFisher Scientific, Waltham, MA, USA). The dialyzed supernatant was passed through a Millipore membrane filter with 0.45 µm pore size (Merck) before being subjected to standard immobilized metal ion affinity chromatography (IMAC) procedure. Chromatography was performed in an ÄKTA purifier FPLC system (GE Healthcare, Danderyd, Sweden) using a 1 mL HisPur^TM^ Ni-NTA chromatography cartridge (ThermoFisher Scientific) as described by the manufacturer with some modifications. Briefly, 75 mL of sample was mixed in a 1:1 proportion with binding buffer (20 mM PBS, 300 mM NaCl, 0.5 mM imidazole, pH 7.5) and loaded at a flow of 1 mL/min into the pre-equilibrated column. The column was washed with 10 mL of washing buffer (20 mM PBS, 300 mM NaCl, 5 mM imidazole, pH 7.5) and the immobilized protein was eluted using an imidazole gradient (5–300 mM imidazole in 40 min) at 0.5 mL/min. The purified fraction was dialyzed overnight at 4 °C against 1 L of PBS pH 8.0 to remove imidazole using a Slide-A-Lyzer™ Dialysis Cassettes (7.5 kDa MWCO) (ThermoFisher Scientific) and 1 mM PMSF protease inhibitor was added. The BCA kit was used to obtain the concentration of the recombinant protein in the purified fraction and calculate the expression yield. The purification step was evaluated by SDS-PAGE by analyzing 10 µL of the supernatant prior and after the IMAC procedure and 10 µL of a 5-fold dilution of the purified dAb8E (2 µg of protein). Finally, the mass spectra of the intact protein was obtained by MALDI-TOF-MS (Ultraflex workstation equipped with a 337 nm nitrogen laser, Bruker Daltonics, Bremen, Germany) at the Mass Spectrometry Unit of Complutense University of Madrid (Spain). One microliter of a 10-fold dilution of the purified antibody was mixed with 1 µL of matrix (10 mg of sinapinic acid in 0.1% trifluoroacetic acid, 30% (*v*/*v*) acetonitrile in water). Measurement was performed in linear positive mode operating in a range between 4000–21,000 *m*/*z*. After a delayed extraction time of 150 ns, ions were accelerated with a 25 kV voltage and data collected from 200 laser shots. FlexControl Software version 2.4. (Bruker Daltonics, Bremen, Germany) was used for sample analysis and control of analytical method parameters.

#### 2.5.3. Protein Identification by Matrix-Assisted Laser Desorption/Ionisation-Time of Flight Mass Spectrometry

The bands of the SDS-PAGE gel obtained from the purified dAb8E were manually excised with a sterile scalpel. Following the protocol described by Sechi and Chait [30] samples were in-gel reduced, alkylated and digested with trypsin. After overnight trypsin digestion, 1 µL of the supernatant was let to dry spotted onto a MALDI plate employing α-cyano-4-hydroxy-cinnamic acid matrix (Sigma) in 50% acetonitrile, and peptides analyzed at the Proteomics Unit of Complutense University of Madrid (Spain) using a 4800 Plus Proteomics Analyzer MALDI-TOF/TOF (Applied Biosystems, MDS Sciex, Toronto, ON, Canada). Protein identification by Peptide Mass Fingerprinting (PMF) was performed in MASCOT v2.6.2 search engine through Global Protein Server (GPS) v.3.6 (ABSCIEX) and search parameters: carbamidomethylcysteine as fixed modification and oxidised methionine as variable modification, one missed trypsin cleavage site allowed, peptide mass tolerance 80 ppm and MS/MS fragments tolerance 0.3 Da.

### 2.6. Assessment of ELISA Methods Based on the Purified dAb8E for the Detection of Gluten in Foodstuffs

#### 2.6.1. Preparation of Protein Extracts and Reference Materials

Firstly, 50 g of each tested sample were individually ground with an IKA A11 analytical mill (IKA^®^, Staufen, Germany), ensuring thorough cleaning between samples, and stored at −20 °C. Protein extracts were obtained from 250 mg of the homogenized samples. Briefly, 2.5 mL of the Ingezim Gluten extraction solution (Ingenasa, Madrid, Spain) was added to the sample, homogenized by vortexing and incubated for 40 min at 50 °C. After cooling the sample to room temperature (RT), 7.5 mL of 80% ethanol was added and the prolamin fraction was solubilized by shaking the tube in a vertical rotator for 1 h. Finally, after centrifugation at 3000× *g* for 10 min at 25 °C, the supernatant was transferred to a fresh glass vial and stored at RT in the dark until further analysis.

In the case of the CRF-INIA cultivars used as reference samples for the gluten-specific detection system, those from the same cereal type were proportionally mixed to obtain three individual reference mixtures of wheat, barley and rye. Extracts were obtained as specified above and the bicinchoninic acid (BCA) assay (Thermo Fisher Scientific Inc., Rockford, IL, USA) was used to quantify protein concentration. The amount of protein in the extracts was normalized to 25 µg/mL and serial dilutions were included in the analyses.

To assess the sensitivity of the assay, the gliadin reference material of the Prolamin Working Group (PWG) (Hans-Dieter-Belitz-Institut, Freising, Germany) was analyzed to obtain a calibration curve. PWG-gliadin stock solution (2 mg/mL) in 60% ethanol was prepared, aliquoted in glass vials stored at RT and used to prepare gliadin dilutions in every analysis. Additionally, the ability of the soluble dAb8E to detect gluten proteins in a real food matrix was evaluated by analyzing experimental binary mixtures prepared as described by García-García et al. 2020 [18]. Briefly, a wheat/barley/rye mixture (WBRm) which contained one third of wheat, barley and rye was employed to prepare rice-based spiked mixtures including different concentrations of WBR (0, 0.1, 0.2, 0.5, 1, 5 and 10 mg/g).

#### 2.6.2. Indirect Enzyme-Linked Immunosorbent Assay (ELISA)

To evaluate the capacity of the purified dAb8E to recognize its target protein, an indirect ELISA was performed against serial dilutions of the PWG-gliadin reference material in the range of 0.1 to 100 µg/mL. One hundred microliters of the standard protein dilutions in 50 mM carbonate-bicarbonate buffer (pH 9.6) was used to coat a 96-multi-well polystyrene plate (F96 MaxiSorp, Nunc immune plates, Nunc, Denmark). The plate was incubated for 1 h at 37 °C and washed 10 times with PBS before being blocked with 200 µL of 2% skimmed milk powder in PBS (M‑PBS) for 1 h at room temperature. After another washing step, 100 µL of the purified dAb8E antibody diluted at 20 µM in carbonate buffer with 3% BSA was added to each well and the plate was incubated at room temperature for 2 h at 750 rpm. After incubation, the plate was washed 10 times with PBS and 100 μL of a 1:5000 dilution of anti-HisTag-HRP antibody in blocking buffer was added to each well and incubated at room temperature for 1 h at 750 rpm. The plate was washed and 100 μL tetramethylbenzidine substrate solution (TMB) added for color development. After incubating for 15 min in the dark with shaking, 50 μL of 1 M sulfuric acid was added and signal intensity measured at 450 nm using a FLUOstar OPTIMA microplate reader (BMG Labtech, Offenburg, Germany). Uncoated wells were included in every analysis to avoid false positive results and the possibility of cross-reactivity of the secondary antibody with the target protein was excluded. Each sample was analysed in triplicate.

Additionally, to determine the potential of the soluble dAb8E to recognize gluten from their natural source, ELISA experiments were performed with ethanolic extracts from cereal samples. The home-made WBRm was selected as the target flour, and commercially available maize, oats and rice flours certified as gluten free were used as non-gluten-containing heterologous species to evaluate the specificity of the assay. ELISA analyses were undertaken as described above with slight modifications: immunoplates were coated with a 1:10 dilution of the flour extracts in carbonate buffer and after the blocking step, soluble dAb8E was added at different concentrations ranging from 5 to 50 µM.

#### 2.6.3. Sandwich Enzyme-Linked Immunosorbent Assay (ELISA) with Phage-dAb8E as Detection Antibody

For the development of the sandwich ELISA used to analyze the commercial food samples, the immunoplates were coated for 1 h at 37 °C with 100 μL of the purified dAb8E diluted at 20 µM in carbonate buffer as capture antibody. After washing 10 times with PBS, the plates were blocked with 200 µL of 2% M‑PBS for 1 h at RT before adding the protein extracts of the WBRm binary mixtures and commercial food products, diluted 1:5 in carbonate buffer. Alternatively, immunoplates were coated with 100 μL of PWG-gliadin and target cereals extracts dilutions at the concentrations specified in each experiment for the construction of dose-response curves. Detection of immobilized gluten proteins was performed with 100 μL of PEG-NaCl purified phage-dAb8E particles in 3% BSA in carbonate buffer (approximately 4·10^11^ phage particles per well). After incubation for 1 h at RT plates were further incubated in the same conditions with 100 μL of anti-M13 monoclonal mouse antibody/HRP (SinoBiological, Chesterbrook, PA, USA) diluted 1:5000 in M-PBS. Color development and absorbance measurement were carried out as indicated above. 

#### 2.6.4. Assay Validation

To determine the potential of the dAb8E antibody to simultaneously recognize gluten from the three main gluten-containing cereals, the obtained mixtures from the kernels of the same cereal types (wheat, barley and ry) were extracted and analyzed in the concentration range 0.2–25 μg/mL. A four-parameter logistic equation was used to fit the concentration-response curves as previously described by de la Cruz et al. (2013) [31]. Subsequently, the PWG-gliadin reference material was assessed in the range 0.1–10 μg/mL and absorbance values fitted to a linear regression. The 4P-logistic model was further used to obtain the correlation curve from the analysis of the rice-based binary mixtures containing 0.1 to 10 mg/g of the WBRm. All the fitting models and correlation coefficients were obtained with Origin 8.0 software (OriginLab Corp., Wellesley Hills, MA, USA). Furthermore, the limits of detection (LOD) and quantification (LOQ) were calculated, respectively, as three and ten times the standard deviation of ten replicates of the blank, respectively [32]. The dilution buffer for the PWG-gliadin linear regression and the protein extract of the un-spiked matrix were selected as the blank sample for the determination of both limits.

The applicability of the developed sandwich ELISA was assessed through the analysis of 30 commercial food products and the obtained results were compared to those generated by means of the monoclonal R5 antibody. For this purpose, the INgezim Gluten Quick kit commercialized by Eurofins (Ingenasa, Madrid, Spain) was employed following the enclosed manual. As indicated by the manufacturer, sample dilution of the commercial food products was set at 1:12.5. This immunoassay presents a LOD of 3 mg/kg of gluten and has been approved as Type I method for gluten analysis in foods by Codex Alimentarius.

## 3. Results and Discussion

### 3.1. Vector Construction

**Figure 1:**
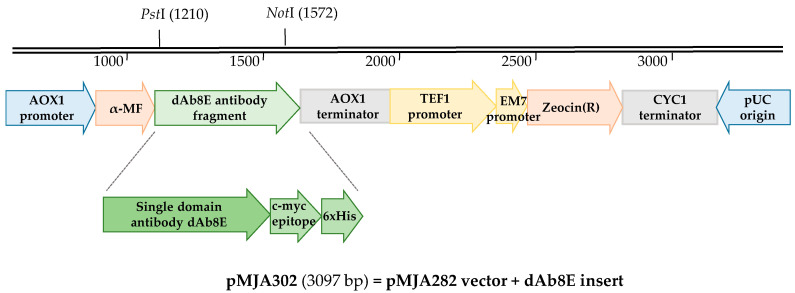
Scheme of the plasmid pMJA302 engineered by inserting the coding sequence of the single-domain antibody (sdAb) fragment dAb8E in the acceptor vector pMJA282. This plasmid was used to produce the recombinant antibody dAb8E against gluten by means of the *Pichia pastoris* expression system. The single-domain antibody fragment (dAb8E) sequence was cloned in frame with the secretion signal pre-pro-region α-mating factor (α-MF) to be produce in the culture supernatant.

Expression vector pMJA282 (in house-build from the commercially available pPICzαB) was used as acceptor plasmid to clone a single-domain antibody fragment against gluten, denominated dAb8E, isolated in our previous work by phage display technology [18]. The ImMunoGeneTics information system for immunoglobulins (IMGT) software (http://www.imgt.org) results from the analysis of the sdAb sequence revealed a mismatched in the 5′‑end of the antibody framework when compared with the database human VH3-23 sequences. Therefore, forward primer MJA448 used to amplify the antibody fragment open reading frame (ORF) was designed to mutate the first amino acid codon from CAG to GAG (turning glutamine in the original phage library sequences into glutamic acid). The resulting vector pMJA302 (Figure 1) is under the control of the AOX1 promoter, one of the most popular promoters for heterologous protein expression in *P. pastoris* due to its remarkably strength and tight methanol regulation, thus the expressed sequence was integrated into the yeast genome via homologous recombination [20]. Besides, the recombinant antibody protein was inserted in frame with the pre-pro region α-mating factor (α-MF), a secretion signal sequence from *Saccharomyces cerevisiae* widely employed to produce the recombinant proteins in the supernatant of the cell culture. During expression, this region will be cleaved by the Kex2 protease after the dibasic amino acids KR and the adjacent EA dipeptides will subsequently be removed by dipeptidyl aminopeptidase A [33].

*E. coli* DH5α was used as host cell for the constructed vector and the insertion of the DNA confirmed by colony PCR. Cell-transformed plasmid vectors were purified and sequenced to confirm its correct identity before being transformed into *P. pastoris* X-33 for the induction of protein expression. The recombinant antibody contained a c-myc epitope (EQKLISEEDL) and a hexa-histidine sequence in the C-terminus that enabled further identification and purification by ion metal affinity chromatography. The coding sequence of the dAb8E antibody fragment, along with the translated amino acids sequence, were uploaded to GenBank with the accession number MW030686.

### 3.2. Expression and Purification of Soluble Antibody Fragments dAb8E

**Figure 2:**
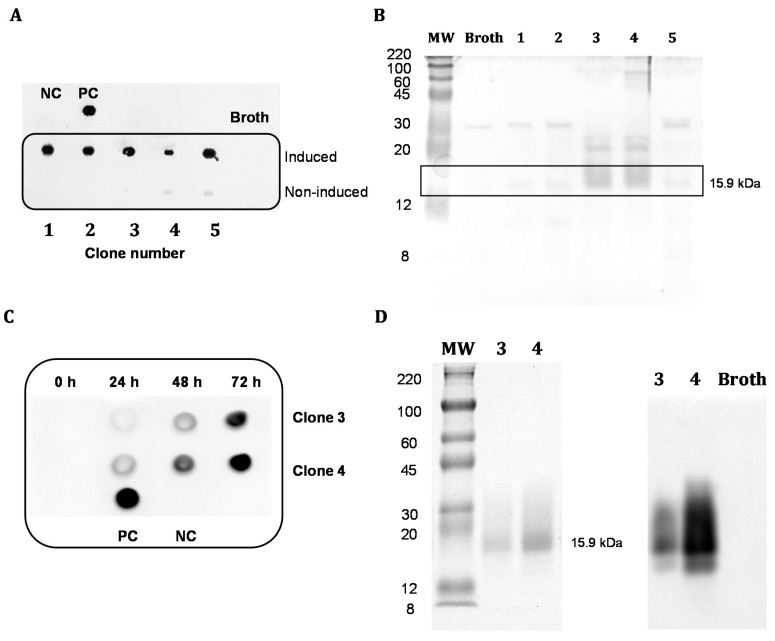
(**A**) Dot-blotting analysis of the small-scale production of five clones of *P. pastoris* transformed with pMJA302 (100 µL of the induced supernatants) revealed with a mouse monoclonal anti-c-myc antibody. The non-induced supernatants, broth media and PBS (NC) were analyzed as negative controls, and 1 µg of a previously purified scFv antibody fragment containing the c-myc epitope was included as positive control (PC). (**B**) Sodium dodecyl sulfate-polyacrylamide gel electrophoresis (SDS-PAGE) electrophoresis (under reducing conditions) of 10 µL of the above induced yeast cultures (lanes 1–5). (**C**) Dot-blotting analysis of the supernatants of two selected clones (clone 3 and 4) at 0, 24, 48 and 72 h of induction (10 µL of a 10-fold dilution of the supernatants). The PVDF membrane was revealed with a rabbit polyclonal anti-HisTag-HRP antibody. (**D**) SDS-PAGE (**left**) and Western Blot analysis (**right**) of 10 µL of the supernatants from clones 3 and 4 transformed with pMJA302 (lanes 3 and 4). The transferred proteins were revealed with the anti-HisTag-HRP antibody. In all cases MW (molecular weight marker) corresponds to the ColorBurst Electrophoresis Marker (Sigma-Aldrich).

After verification by colony PCR, the small-scale production of five clones positively transformed with pMJA302 were analyzed by dot-blotting and SDS-PAGE (Figure 2A,B). As can be observed, membrane incubation with an anti-c-myc-antibody revealed a high color intensity in the supernatants after 72 h of methanol induction compared to the non-induced clones, thus indicating the proper expression of soluble proteins in the culture media. To further evaluate the expression of the single-domain antibody fragment, the supernatants were subjected to SDS-PAGE analysis which revealed that only clones 3 and 4 showed a band with an apparent molecular weight near to the theoretical molecular mass of 15.95 kDa for the sdAb fragment. In light of these results, the production from these two selected clones was scaled-up and the expression in the culture medium monitored over time. As shown in Figure 2C, after 24 h of induction the presence of recombinant protein in the supernatant was remarkable, increasing the signal intensity over time up to 72 h. Apparently, the expression level of clone 4 was significantly higher than that of clone 3, which was confirmed by SDS-PAGE and western blot analysis of the 72 h-supernatants with a secondary anti-HisTag antibody (Figure 2D). These results indicated that clone 4 transformed with vector pMJA302 exhibited a high production of a recombinant protein at the expected size, both by gel staining and immunoblotting, which could be positively detected by means of c-myc and HisTag epitopes. Therefore, the supernatant of this specific clone was subsequently subjected to immobilized metal ion affinity chromatography (IMAC) procedure for the purification and further characterization of the recombinant antibody fragment.

**Figure 3:**
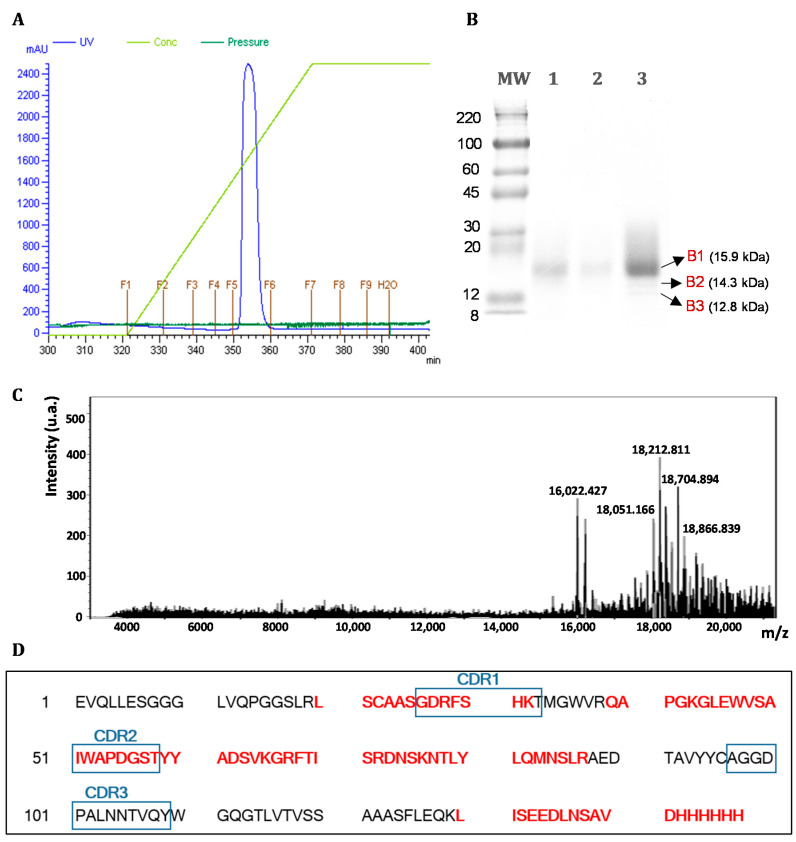
(**A**) Immobilized metal affinity chromatography (IMAC) profile for the purification of the recombinant dAb8E from the supernatant of clone 4 transformed with vector pMJA302. (**B**) Analysis of the purification steps by SDS-PAGE electrophoresis (under reducing conditions). Lanes 1 and 2 correspond respectively to the analysis of 10 µL the induced supernatant prior and after to purification by means of IMAC, and lane 3 correspond to the analysis of the main purification peak (fraction 5) after the dialysis step (2 µg of protein). The indicated bands were excised, trypsinized and the peptide mass fingerprinting analysis performed. MW (Molecular marker) corresponds to ColorBurst Electrophoresis Marker (Sigma-Aldrich). (**C**) MALDI-TOF mass spectra of the intact dAb8E produced in *P*. *pastoris*. (**D**) Mass fingerprinting analyzed from band 1 (B1): matched peptides from MS spectra after Mascot search are represented in red bold font in the amino acids sequence of the dAb8E (GenBank accession number MW030686). The dAb8E complementarity determining regions (CDR1-3) are highlighted in blue boxes.

Antibody fragments produced as hexahistidine-tagged protein in *P. pastoris* were purified from the clarified supernatant by IMAC chromatography with an imidazole gradient as the elution buffer. As observed in the chromatographic profile shown in Figure 3A, the concentration of imidazole that eluted the main protein fraction retained in the column was approximately 200 nM. The analysis by SDS-PAGE of the supernatant prior and after the IMAC purification step showed that the retention efficiency of the column employed for purification was satisfactory (Figure 3B). Besides, in order to confirm the obtaining of the desired antibody fragment with acceptable purity and homogeneity, the purified fraction was dialyzed to remove imidazole before being subjected to MALDI-TOF analysis. The mass spectra obtained from the analysis of the intact protein is shown in Figure 3C. The experimental mass of the sdAb was in good agreement with the one calculated from the full amino acids sequence (15,952 Da; +70 Da mass difference). Peaks observed at *m*/*z* values above 17,000 were characterized by a distribution of equidistant peaks (162 Da increment), which according to other authors could correspond to the addition of hexose (e.g., mannose) due to the ability of *P. pastoris* to generate post-translational modifications such as glycosylation [34,35]. According to this result, the recombinant antibody was purified to substantial homogeneity.

Three independent bands cut-out from lane 3 of the SDS-PAGE gel of Figure 3B were subjected to tryptic digestion to further confirm the identity of the purified protein. The peptide mass fingerprinting analysis was identified in all the samples by Mascot engine as *Homo sapiens* partial IGHV3-23 gene for immunoglobulin heavy chain variable region. In all the peptides identification, the probability scores were greater than the score fixed by Mascot as significant with a p-value minor than 0.05 (cut-off). For the analysis of the main band of the purified dAb8E (B1 at 15.9 kDa), the obtained Mascot score was of 102 being the cut-off value 13, which demonstrated the high reliability of the identification. This fact was consistent with the high percentage of coverage of the theoretical amino acids sequence calculated as 54% (Figure 3D). Furthermore, it was observed that the identified peptides covered the complementarity determining regions CDR1 and CDR2, thus evidencing the expression of the gluten-specific antibody fragment dAb8E. The presence of protein bands at lower molecular weights (B2 and B3) might be indicative of antibody-degradation products along the purification stages, since the dAb8E sequence is still identified but at slightly lower coverages of 51%. In this sense, other authors have reported that pH value of the induction media is a critical point when VHH antibody fragments are produced, observing protease activity for pH values above 6 and hydrolysis degradation if pH drops below 5 [36]. Thus, and despite the fact that in the present work the buffered induction medium was kept at pH 6 and a protease inhibitor was added, the presence of a small proportion of antibody degradation products was observed. However, this observation did not hamper the proper functionality of the antibody in the development of a sandwich ELISA for the analysis of gluten in foods.

The concentration of the purified dAb8E was calculated as about 8.6 mg/mL by the BCA’s method. Therefore, the obtained production yield was 330 mg/L for the secretion of a single-domain antibody fragment using *P. pastoris* as expression system. Due to the minimal release of endogenous proteins into the culture medium of this yeast, the secretion of the recombinant protein is a very attractive alternative, because it allows simple growth under aerobic conditions and facilitates subsequent protein purification [37]. Similar works have reported fewer yields of 10–15 mg/L [36] and 25 mg/mL [38] for the production of functional sdAb and scFv, respectively, in *P. pastoris*, evidencing the high-yield production of the selected clone.

### 3.3. Development of an ELISA Methodology for the Analysis of Gluten in Foods

**Figure 4:**
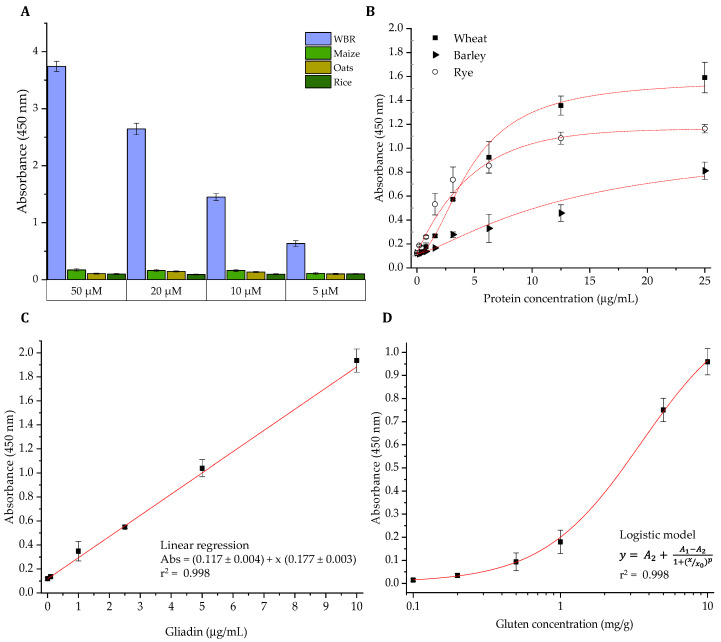
Assessment of a immunoassay techniques based on the purified dAb8E (**A**) Comparison of dAb8E binding by indirect ELISA to extracts WBRm, maize, oats and rice at the specified antibody concentrations (WBRm stands for a representative mixture of a wheat, barley and rye kernels) (**B**) Sandwich ELISA (soluble dAb8E and phage-dAb8E as the capture and detection antibodies, respectively) for the analysis of individual reference mixtures (40 wheats, 10 barleys and 10 ryes) at different protein concentration levels (0.1 to 25 µg/mL) (**C**) Linear regression obtained from the absorbance values of the analysis of 0.1–10 µg/mL PWG-gliadin by dAb8E-based sandwich ELISA. (**D**) Four-parameter logistic curve obtained from the analysis of binary mixtures consisting of rice flour experimentally spiked with decreasing amounts of WBRm (0.1–10 mg/g by dAb8E-based sandwich ELISA. This model was used to interpret the gluten content of the tested commercial food products. These fitting models have been obtained with Origin 8.0 software and represents the average values and standard deviations of one representative experiment performed in triplicate.

Gluten-specificity of the dAb8E was extensively studied in our previous work by testing ethanolic extracts from a wide representation of heterologous plant species [18]. In order to corroborate these results, an indirect ELISA based on the purified dAb8E was employed to analyze immunoplates coated with a mixture of wheat, barley and rye (WBR) as the target specie and maize, oats and rice flours as heterologous species (Figure 4A). As expected, no cross-reactivity of the soluble antibody with the other cereals was observed despite the phylogenetic closeness of the selected species. Besides, soluble dAb8E bound to WBR proteins in a concentration-dependent manner, proving the gluten specificity of the antibody fragment.

Sandwich ELISA remains the “gold standard” immunoassay test regarding the analysis of allergens in foods since it allows the study of complex samples. This is mainly due to the ability of the coating antibody to specifically bind the antigen from the crude sample extracts. Besides, the use of an antibody pair increases the specificity of the assay. Despite the undeniable advantages of phage display technology in antibody selection, the development of sandwich ELISA requires the generation of the soluble antibody fragment to be used as coating antibody. For this purpose, *P. pastoris* was used to produce the soluble dAb8E selected as the capture antibody due to its small size and high stability in the presence of denaturing agents like those used in gluten extraction. Phage-dAb8E was chosen as the detection antibody for its strong detectability employing a secondary antibody against the major coat protein of the phage (pVIII). Figure 4B illustrates the curves obtained from the study of serial dilutions of extracts from individual mixtures of wheat, barley and rye grain cultivars in the protein concentration range of 0.2–25 µg/mL. The single-domain antibody successfully recognized the three *Triticeae* tribe cereals responsible for triggering celiac disease, although the response towards wheat and rye gluten was considerably stronger compared to the detection of barley proteins, especially at low protein concentrations.

The sensitivity of the produced single-domain antibody was firstly assessed through the analysis of the PWG-gliadin reference material (Figure 4C). The linear dynamic range of the assay was from 0.1 to 10 µg/mL and the detection limit was calculated as 0.12 µg/mL from the interpolation of three times the deviation of the blank. Subsequently, as immunoassay methodologies might be frequently influenced by matrix effects, sensitivity of the sandwich ELISA was further evaluated through the analysis of rice-based binary mixtures [39,40]. Figure 4D shows a representative dose response curve constructed from the analysis of rice flour spiked with decreasing amounts of the wheat/barley/rye mixture (WBRm) at 0.1–10 mg/g. On the basis of these results, the limits of detection and quantification of the sandwich ELISA for the detection of the target cereals in a real food matrix were calculated as 0.13 and 0.31 mg/g, respectively. According to other authors, the gluten level of wheat might be estimated as an overall of about 10% of the cereal weight [41]. Adopting this theoretical approach, the developed ELISA would have the capacity to detect levels of wheat, barley and rye corresponding to gluten concentrations of approximately 13 mg/kg. Therefore, although with limited sensitivity, the dAb8E-based ELISA could be used to verify compliance with gluten labelling regulations and to ensure the access of sensitive consumers to safe products. 

### 3.4. Comparative Quantification of Gluten in Commercial Food Products

The potential of the developed ELISA to determine gluten in a wide representation of different matrices was assessed by analyzing 30 commercial food products divided in four different categories regarding their labelling information (Table 1). In order to validate the results obtained with the developed assay, gluten content of the samples was also evaluated by means the official method for gluten detection in foods, consisting in a sandwich ELISA based on the R5 monoclonal antibody., The optimal sample dilution for the developed immunoassay was set at 1:5, which contrasts with the minimum dilution required for the use of the mAbR5 of 1:12.5. This result is in agreement with the higher stability of the single-domain antibodies in the presence of ethanol and denaturing agents in comparison to conventional immunoglobulins.

As observed in Table 1, the comparison of the results obtained with both methodologies showed good agreement. The gluten levels determined enabled the classification of the samples as suitable or unsuitable for consumption by sensitive individuals regarding the established limit of 20 mg/kg of gluten. Four samples (rice cake, seeds, oats beverage 1 and oats bran) produced differing results, most of which might be explained by the different sensitivity of both immunoassays. In these samples, the official method reported gluten levels in the range of 13.1–15.6 mg/kg that were not detectable with the developed assay due to its closeness to its detection limit.

To the best of our knowledge, only the work by Doña et al. (2010) has reported the production of a single-domain antibody fragment intended for gluten determination in foods [17]. Similarly to the present work, the recombinant antibodies reported by these authors demonstrated excellent performance allowing the analysis of samples at lower dilutions which increased the sensitivity of the assay. However, these antibodies raised in immunized llamas failed to recognize protein extracts from barley and rye, being only able to detect gluten from wheats. In addition, the ELISA methodology implemented in the present work is relied on a gluten-specific antibody fragment obtained by García-García et al. (2020) bypassing the use of animal immunization, hence adhering to the latest animal welfare policies.

In conclusion, the present work describes the high-yield production of a recombinant single-domain antibody (dAb8E) by the yeast *Pichia pastoris* and the development of a sandwich ELISA methodology for the analysis of gluten in foods. The purified dAb8E antibody maintained its ability to detect gluten from the three main gluten-containing cereals (wheat, barley and rye) and enabled gluten determination in a broad representation of processed food matrixes. However, the immunoassay developed in the present work was not able to reach the limits of detection of other commercially available ELISA kits. In this aspect, since the recombinant antibody was isolated by phage display technology, the availability of the codifying nucleotide sequence will enable the improvement of the assay in future works. In this sense, the use of in vitro affinity maturation methodologies or the engineering of larger immunoglobulin molecules such as tandem-dAbs or Fabs could provide new alternatives for the generation of new gluten-specific biorecognition molecules.

## Figures and Tables

**Table 1:** Comparison of the results obtained for the detection of gluten in the analysis of 30 commercial food products using the developed sandwich-dAb8E enzyme-linked immunosorbent assay (ELISA) and the official method based on mAbR5 antibody.

	Sandwich dAb8E	Sandwich mAbR5
(A) Category of products that declared wheat, barley, rye and/or gluten in their labelling		
Breakfast cereal 1	>100	>100
Breakfast cereal 2	>100	>100
Chocolate bar	>100	>100
Bread 1	>100	>100
Bulgur	>100	>100
Soup	>100	>100
(B) Category of products that declared may contain traces of wheat, barley, rye and/or gluten		
Rice cake	<LOD	13.1 ± 0.2 *
Pre-cooked meal	<LOD	<LOD
Snack 1	>100	>100
Seeds	<LOD	13.5 ± 0.4
Breakfast cereal 3	27 ± 0.3	26.4 ± 0.3
(C) Category of products that did not declare wheat, barley, rye nor gluten in their labelling		
Breakfast cereal 4	>100	>100
Breakfast cereal 5	23.9 ± 0.2	28.2 ± 0.7
Breakfast cereal 6	28.6 ± 0.1	77.5 ± 0.5
Oats beverage 1	<LOD	15.6 ± 0.1
Oats beverage 2	<LOD	<LOD
Oats bran	<LOD	14.3 ± 0.3
(D) Category of products certified as gluten free		
Breakfast cereal 7	<LOD	<LOD
Nut bar	26.5 ± 0.3	35.2 ± 0.2
Bread 2	<LOD	<LOD
Bread 3	<LOD	<LOD
Biscuit 1	<LOD	<LOD
Biscuit 2	<LOD	<LOD
Snack 2	<LOD	<LOD
Pasta 1	<LOD	<LOD
Pasta 2	<LOD	<LOD
Pasta 3	<LOD	<LOD
Pasta 4	<LOD	<LOD
Flour	<LOD	<LOD
Bakery	<LOD	<LOD

Sandwich dAb8E: sandwich ELISA based on the purified single domain antibody dAb8E against gluten; Sandwich mAbR5: commercially available sandwich ELISA based on the monoclonal antibody R5; * Mean value and standard deviation of three independent measurements (n = 3); <LOD: acquisition of absorbance values (450 nm) under the limit of detection (LOD) of the assays (13 and 3 mg/kg for dAb8E-based and mAbR5-based sandwich ELISA, respectively).

## Appendix A

### Appendix A.1. Procedure for Obtaining Escherichia coli Competent Cells

Firstly, 100 mL LB media was inoculated with an overnight culture and grown at 37 °C until OD_600_ reached 0.4. Culture was cooled down to 4 °C on ice for 30 min and this temperature was strictly maintained in all the following steps. Cells were harvested by centrifugation at 3000 g for 15 min and further resuspended in 50 mL of 100 mM ice cold MgCl_2_. Another centrifugation step was carried out at 2000 g for 15 min and the pelleted cells were resuspended in 50 mL of 100 mM ice cold CaCl_2_. The suspension was incubated on ice for 20 min before being centrifuged at 2000 g for 15 min and the cells resuspended in 50 mL of ice cold 85 mM CaCl_2_ with 15% glycerol (*v*/*v*). Finally, the cells were harvested by centrifugation at 1000 g for 15 min and resuspended in 2 mL of 85 mM CaCl_2_ with 15% glycerol (*v*/*v*).

### Appendix A.2. Procedure for Obtaining Pichia pastoris Electrocompletent Cells

An overnight culture was used to inoculate 250 mL of YPD and incubated at 30 °C with vigorous shaking (300 rpm) until OD600 was 1.5. At this moment, the culture was cooled down on ice, centrifuged at 3000 g for 5 min at 4 °C and the pellet resuspended in 50 mL YPD. After adding 1.25 mL 1 M dithiothreitol (DTT), the culture was incubated at 30 °C for 15 min and 200 mL of ice-cold 1 M sorbitol (icS) was added. Cells were pelleted at 3000 g for 5 min at 4 °C, resuspended in 50 mL icS and centrifuged again under the same conditions. Yeast cells were resuspended in 2 mL icS, pelleted in the same centrifuge conditions and finally resuspended in 250 µL of icS and kept at 4 °C to be immediately used for electroporation.
